# High resolution mapping and positional cloning of ENU-induced mutations in the *Rw *region of mouse chromosome 5

**DOI:** 10.1186/1471-2156-11-106

**Published:** 2010-11-30

**Authors:** Yung-Hao Ching, Robert J Munroe, Jennifer L Moran, Anna K Barker, Evan Mauceli, Tim Fennell, Frederica diPalma, Kerstin Lindblad-Toh, Lindsay M Abcunas, Joyanna F Gilmour, Tanya P Harris, Susan L Kloet, Yunhai Luo, John L McElwee, Weipeng Mu, Hyo K Park, David L Rogal, Kerry J Schimenti, Lishuang Shen, Mami Shindo, James Y Shou, Erin K Stenson, Patrick J Stover, John C Schimenti

**Affiliations:** 1 Department of Biomedical Sciences, College of Veterinary Medicine, Cornell University, Ithaca, New York 14853, USA; 2 The Broad Institute, Seven Cambridge Center, Cambridge, MA 02142, USA; 3 Division of Nutritional Sciences, Cornell University, Ithaca, New York 14853, USA; 4 Science for Life Laboratory, Department of Medical Biochemistry and Microbiology, Uppsala University, Box 582, SE-751 23 Uppsala, Sweden

## Abstract

**Background:**

Forward genetic screens in mice provide an unbiased means to identify genes and other functional genetic elements in the genome. Previously, a large scale ENU mutagenesis screen was conducted to query the functional content of a ~50 Mb region of the mouse genome on proximal Chr 5. The majority of phenotypic mutants recovered were embryonic lethals.

**Results:**

We report the high resolution genetic mapping, complementation analyses, and positional cloning of mutations in the target region. The collection of identified alleles include several with known or presumed functions for which no mutant models have been reported (*Tbc1d14*, *Nol14*, *Tyms*, *Cad*, *Fbxl5*, *Haus3*), and mutations in genes we or others previously reported (*Tapt1*, *Rest*, *Ugdh*, *Paxip1*, *Hmx1, Otoe, Nsun7*). We also confirmed the causative nature of a homeotic mutation with a targeted allele, mapped a lethal mutation to a large gene desert, and localized a spermiogenesis mutation to a region in which no annotated genes have coding mutations. The mutation in *Tbc1d14 *provides the first implication of a critical developmental role for RAB-GAP-mediated protein transport in early embryogenesis.

**Conclusion:**

This collection of alleles contributes to the goal of assigning biological functions to all known genes, as well as identifying novel functional elements that would be missed by reverse genetic approaches.

## Background

Since the human and mouse genomes were sequenced several years ago, it has been recognized that the next major challenge is to uncover the functional content of the genome. This was a motivation behind large scale projects such as ENCODE (Encyclopedia of DNA elements) and KOMP (Knockout Mouse Project; http://www.nih.gov/science/models/mouse/knockout/komp.html). The former used molecular strategies to identify features of human chromatin [1], and the next iteration (modENCODE) is geared to identifying functional genomic elements *in vivo *using non-vertebrate models http://www.genome.gov/26524507. KOMP uses a reverse genetic approach to identify the functions of all known and annotated mouse protein coding genes, by generating germline or conditional null alleles in embryonic stem (ES) cells.

Forward genetic mutagenesis in mice is most commonly conducted via whole animal mutagenesis with the point mutagen *N-ethyl-N*-nitrosourea (ENU)[2]. This approach has succeeded in identifying new genes that play important roles in biological processes and in modeling disease [3-8]. Two key advantages over reverse genetics are that: 1) ENU can cause non-null alleles, enabling the dissection of protein function and yielding variants that are more relevant to disease-causing mutations in humans; and 2) as a non-biased approach, genetic elements or genes can be identified that would never have been implicated *ab initio *to have specific activities or roles.

In multiple organisms, point mutagens have been employed in two basic strategies: random and region-directed. The former has the benefit of scanning the whole genome, whereas the latter allows one to focus resources on a microcosm of the genome. Several years ago, we initiated a larger scale regional screen of proximal mouse Chr 5 spanned by an inversion called rump-white (*Rw*). This recessive lethal balancer facilitated the isolation and maintenance of mutations recovered in the screen. Predominantly lethal mutations were recovered (a total of 37) [9]. Here, we report on the mapping and cloning of much of this collection. Several mutations were identified in genes for which no models previously existed, providing new biological insights and models of interest. Intriguingly, we also mapped one mutation to a gene desert, in which the nearest protein-coding gene is over 1 megabase away.

## Results and Discussion

### Mutation Mapping

The rough locations of the *Rw *region ENU-induced mutations were reported by Wilson et al [9], with most of them being localized to intervals that were too large to allow effective candidate gene selection. Additionally, since many of the mutations were located in common large intervals, allelism amongst them was possible. To map the mutations at higher resolution, we continued and expanded upon two strategies initiated by Wilson et al: deletion mapping and recombination mapping [9].

**Figure 1:**
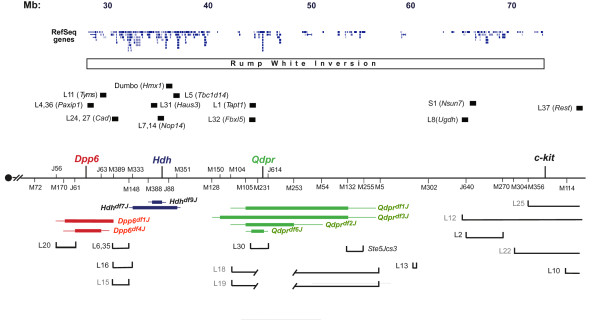
**Map of mutations in *Rw *region**. The proximal region of Chr. 5 is depicted as a horizontal line, with the centromere (filled circle) on the left. The region spanned by the *Rw *inversion is indicated above. Map positions (in megabases, accordingly to mouse genome Build 37) are indicated. Microsatellite loci are abbreviated by exchanging the prefix "M" for "*D5Mit*" and "J" for "*D5Jcs*". Deletions are indicated as horizontal rectangles, and are color coded with the locus at which they were induced (red) *Dpp6*; (blue) *Hdh*; (green) *Qdpr*. The amount of DNA known to be absent in each deletion is spanned by the rectangles. The thin lines extending from the ends of the rectangles indicate the regions in which the deletion breakpoints reside. The intervals containing certain lethal mutations (abbreviated as "L#") are bracketed at the bottom of the map. The end points of the brackets correspond to markers on the map above. Cloned mutations are shown above the map. Uncloned lethal mutations that are no longer extant are greyed out names. The locations of RefSeq genes are indicated at the top. This depiction is a modified screenshot from the UCSC browser. Note that some genes have multiple isoforms, all of which are indicated.

In the deletion mapping approach, complementation tests between nearly all the lethals and relevant *Dpp6*, *Hdh *and *Qdpr *deletions were completed (Figure 1). Various smaller deletions in each complex (e.g. *Dpp6*^*df4J*^, *Hdh*^*df9J*^, *Qdpr*^*df6J *^and others shown in Figure 1) were used to better refine locations of noncomplementing lethals. Importantly, complementation of mutations by deletions also provided useful information by excluding large regions. The map positions of *L5Jcs6*, *15*, *16 *and *24 *were determined in large part by the fact that they are complemented by both *Dpp6*^*df1J *^and *Hdh*^*df7J*^. Recombination mapping of mutations was performed as described in Methods.

The mutation mapping is summarized in Figure 1. Several of the embryonic lethal mutations mapped near or beyond the distal end of the *Rw *inversion, and these lines were not maintained (most are not shown here, but are presented in [9]). Figure 1 and Table 1 also indicate those cases in which the mutated gene has been identified (or likely so). At present, 16 mutants have been identified from the original 37 that mapped within or near the *Rw *inversion region. Not surprisingly, the mutations cluster to regions that are dense in RefSeq gene annotations (Figure 1). An exception is *L5Jcs13*, which maps to gene desert (discussed below).

**Table 1:** Positional Cloning of Mutations Summary

Allele	Gene	Mutation	Genetic Interval	Gene Function	Refs
*L5Jcs1*	Tapt1 **	see ref	see ref	Mutations causes homeotic-like skeletal transformations and perinatal lethality.	[11]

***L5Jcs4 L5Jcs36***	Paxip1 **	see ref	see ref	Vasculogenesis; DNA repair; epigenetic regulation	[10,36]

*L5Jcs5*	*Tbc1d14*	missense in TBC1 domain	M4-J57 (1.6 Mb)	TBC1 domain family; putative RAB GTPase activation protein.	

***L5Jcs7 L5Jcs14***	*Nop14*	SA site, exon 3 nonsense, exon 3	J36-M268 600 kb	Nucleolar protein homolog; ribosome biosynthesis.	

*L5Jcs11*	*Tyms*	T > A (ASN > LYS)	J63-M176 (1.1 Mb)	Thymidylate synthase	
*L5Jcs37*	Rest *	C893G (P > R)		RE1-silencing transcription factor	[37]

*Dumbo*	Hmx1**	see ref	see ref	Homeobox-containing gene	[38]

***L5Jcs24 L5Jcs27***	*Cad*	See ref	M251-M334 M387-M353	carbamoyl-phosphate synthetase 2, aspartate transcarbamylase, and dihydroorotase.	in prep^

*L5Jcs31*	*Haus3*	missense LEU > PRO in exon 4.	M388-J88 (1.8 Mb)	Subunit of augmin (HAUS) complex that regulates centrosome and spindle integrity.	

*L5Jcs32*	*Fbxl5*	missense MET > LYS	M105-J614	Part of E3 ubiquitin ligase complex that regulates iron homeostasis.	[22,23]

*L5Jcs8*	Ugdh *	nonsense	D5Ncnp1-J24 (1.3Mb)	UDP-glucose dehyrogenase; GAG biosynthesis; gastrulation.	[39]

*Ste5Jcs1*	Nsun7 **	see ref	see ref	Required for normal sperm motility and male fertility.	[40]

*Deaf5Jcs1*	Otof **	see ref	see ref	Otoferlin; inner hair cell neurotransmission	[41]

*Separate alleles reported by others. ** Positional cloning of this allele previously reported. M = *D5Mit*; J = *D5Jcs*. Bolded alleles highlight that two alleles were identified. ^Ching & Schimenti *et al *will report on these alleles elsewhere. Alleles beginning with "L" are embryonic lethals; timing of lethality was reported previously [9].

### Complementation analyses of the mutant collection

**Figure 2:**
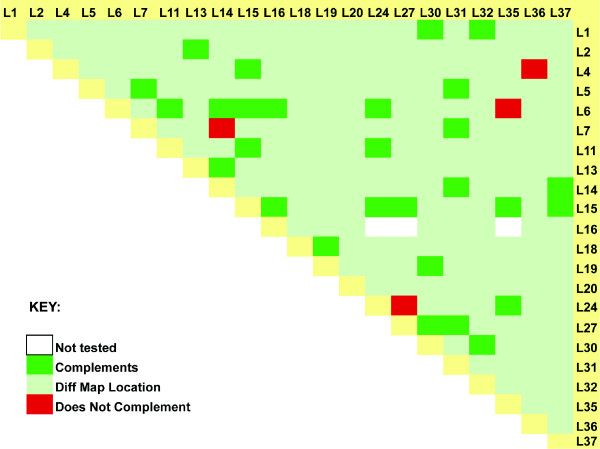
**Complementation and allelism analyses**. The grid shows all pairwise comparisons of *Rw*-region lethal mutations for potential allelism. Original mutations located distal to *Rw *are not included, nor is *L5Jcs17*, which went extinct before mapping and allelism testing could be completed. Actual complementation tests were, in most cases, only performed when mutations co-mapped to overlapping genetic intervals (dark green or red). The majority of mutations were mapped to sufficient resolution to indirectly conclude they are not allelic (light green).

Because ENU mutagenesis is random, some of the mutations may be alleles of the same gene. An allelic series can give insight into protein function. This proved to be the case with *L5Jcs4 *and *L5Jcs36*, alleles of *Paxip1 *(*Ptip*) with differing severity [10]. Furthermore, multiple alleles can solidify evidence that the mutated gene indeed corresponds to the observed phenotype. Accordingly, we evaluated each mutation for potential allelism with the entire mutant collection. Rather than performing complementation tests between all possible pairwise combinations by breeding, we exploited the fact that mutation pairs with non-overlapping map positions cannot be alleles (unless a gene spans adjacent genetic intervals). This greatly reduced the number of potential complementation tests (37 × 36 = 1332) to a subset that co-localized at the time of analysis. Mutations that went extinct before positional cloning and completion of complementation analyses, and most of those that potentially map distal to the *Rw *inversion, are not included. The results are summarized in Figure 2. In sum, we identified allelism between four pairs of mutations: *Paxip1*^*L5Jcs4 *^*/Paxip1*^*L5Jcs36 *^[10], *Cad*^*L5Jcs24*^/*Cad*^*L5Jcs27 *^*(*Ching & Schimenti, in preparation), *Nop14*^*L5Jcs7*^/*Nop14*^*L5Jcs14 *^and *L5Jcs6/L5Jcs35 *(gene not yet identified).

### Positional Cloning of Mutations

Once mutations were mapped to intervals under 5 cM, or to regions containing a small number of annotated genes, we initiated searches for causative mutations. The strategies varied during the course of the project as technology advanced. In most cases, annotated genes were prioritized for mutational analysis by considering the following: 1) phenotypes of described knockouts; 2) whether the genes had orthologs in other species with mutant phenotype information; and 3) gene expression patterns from microarrays or EST library origins. Primer pairs were then designed to amplify exonic sequences from the prioritized candidate genes, using genomic DNA of mutant heterozygotes (or homozygous embryonic material in some cases) as templates. PCR products were analyzed by denaturing HPLC (to detect mismatches as heteroduplexes) or direct sequencing. This led to the identification of several mutations summarized below. The causative nature of the identified mutations is relatively confident in cases where two alleles were identified, or when the mutated gene has a similar knockout phenotype. Otherwise, follow-on confirmation of causality will be required by methods such as non-complementation with a targeted or gene-trap allele (as in the case of *Tapt1*; see below).

The following paragraphs in this section describe positional cloning of new alleles, or relevant additional information on previously identified alleles. Table 1 also overviews all alleles identified in this project.

#### *L5Jcs1*

This perinatal lethal mutation was identified previously as affecting a novel gene, *Tapt1 *(Transmembrane anterior posterior transformation 1) [11]. Homozygous mutants exhibit homeotic transformation of the skeleton. To confirm that the point mutation is causative for the phenotype, we performed a complementation test between *L5Jcs1 *and a mice carrying a deletion allele of *Tapt1 *(*Tapt1*^*KO*^; see Methods). Intercrosses between *Tapt1*^*KO*^/*Rw *and *L5Jcs1*/*Rw *animals produced litters (N = 31 pups) in which only *Rw *offspring (21) survived to wean age. The remaining animals (10) died shortly after birth, before the *Rw *phenotype could be determined. Given that *Rw/Rw *causes embryonic lethality shortly after gastrulation, the expected number of non-*Rw *animals for those surviving to wean age is 7, and the lack thereof is significant (Chi square = 10.5; *P *= .0012). This confirms that the mutation in *Tapt1 *is causative for the *L5Jcs1 *lethal phenotype, and indicates that *Tapt1*^*L5Jcs1 *^is a null or severe hypomorph.

#### *L5Jcs5 (Tbc1d14)*

**Figure 3:**
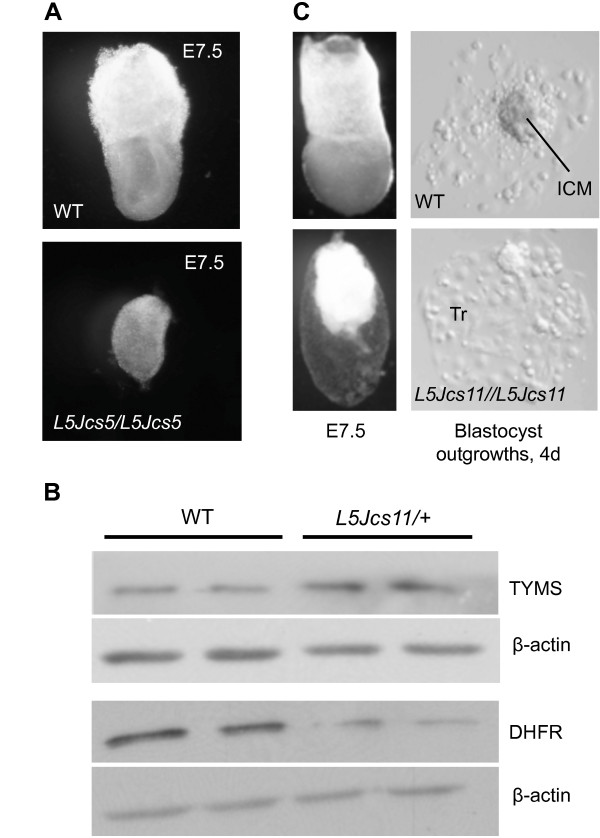
**Phenotypes of *L5Jcs5 *and *L5Jcs11 *mutants**. (A) Light micrographs of E7.5 WT and *L5Jcs5/L5Jcs5 *littermates taken at same magnification (8×). (B) Western blots of liver protein probed with anti-TYMS, DHFR, and beta actin. Each lane contains protein from separate animals. (C) Light micrographs of whole mount E7.5 WT and *L5Jcs11/L5Jcs11 *embryos, plus representative images of blastocyst outgrowths from the indicated genotypes. The mutant embryo is magnified 1.5 X compared to the WT. Notice that there is no evidence of growth of the embryo proper. ICM = Inner cell mass. Tr = trophectoderm.

This mutation was mapped with a combination of F2 crosses and deletion non-complementation. It resides within the *Hdh*^*df7J *^deletion region, but the proximal end was better defined by mapping of recombination breakpoints. It was originally identified as a pre-E9.5 embryonic lethal of undefined phenotype [9]. We performed timed matings to better characterize the developmental phenotype. Embryonic development was disrupted at a very early point post-implantation. At E7.5, when WT embryos have progressed to a late primitive streak stage, mutant embryos are smaller and appear arrested at an egg cylinder-like stage (Figure 3a).

*L5Jcs5 *was mapped to a 1.6 Mb region containing ~17 genes whose knockout phenotypes are not known. Sequencing of several of these genes (not all to completion) revealed only 1 mutation in *Tbc1d14*. This gene encodes a protein containing a TBC (Tre-2/Bub2/Cdc16) domain characteristic of RAB-GAPs, proteins that activate GTPase activity of RAB proteins [12,13]. RABs are guanine nucleotide binding proteins that mediate membrane-associated protein transport. The mutation causes a SER > GLY change in amino acid 433 (NP_001106833; or 413 for NP_598671), which resides in the TBC1 domain. This amino acid is conserved in mammals (dog; human), zebrafish and chickens. There have been no studies regarding the function of TBC1D14 in cells or animals, however the crystal structure of the human protein has been reported [14].

Interestingly, none of the 39 mouse genes annotated as a RAB-GAP (Gene Ontology term: Rab GTPase activator activity; GO:0005097) has been associated with a mutant phenotype of embryonic lethality. Thus, if indeed the *Tbc1d14 *mutation proves to causative for the *L5Jcs5 *phenotype, this would be the first implication of a critical developmental role for RAB-GAP-mediated protein transport in early embryogenesis.

#### *L5Jcs7/L5Jcs14 (Nop14)*

**Figure 4:**
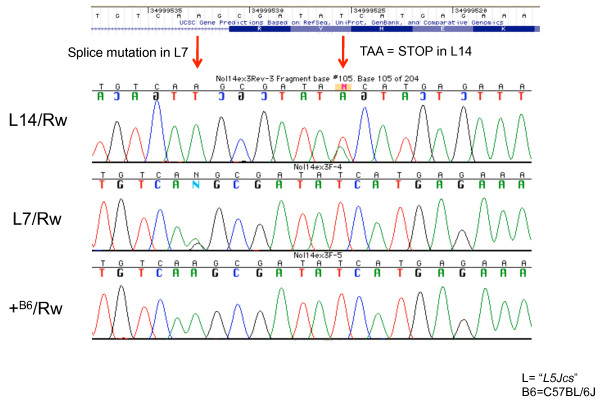
**Mutations in *Nop14***. DNA sequence traces from DNAs of the indicated genotypes, corresponding to 5' end of *Nop14 *exon 3 (a.k.a. *Nol14*), are aligned against the corresponding region from the UCSC gene browser track. The alternating dark/light blue boxes contain the encoded amino acids in exon 3.

Pooled recombination mapping data of these two non-complementing mutations localized the culprit gene to a 600 kb interval containing 13 RefSeq genes. Mutations in *Nop14 *were identified by next-gen sequencing of hybrid-selected exonic sequences (see Methods), and verified by Sanger sequencing (Figure 4). *L5Jcs7 *alters the exon 3 splice acceptor sequence (AG|CG >GG|CG) of this 18 exon gene, whereas *L5Jcs14 *causes a premature stop codon in exon 3. Based on homology to yeast orthologs, this gene is annotated as "nucleolar protein homolog 14," and is involved in ribosome biogenesis [15]. The pre- or peri-implantation lethality caused by these mutations [9] is consistent with such a fundamentally important cellular role.

#### *L5Jcs11 (Tyms)*

*L5Jcs11 *is an early postimplantation mutant overtly defective in gastrulation [9]. It was mapped genetically to a ~1.1 Mb interval by a combination of deletion mapping (*Dpp6*^*df4J *^complemented *L5Jcs11*, defining the proximal end as being distal to *D5Jcs63*) and recombination mapping (defining the distal end of the critical region). Sequencing of candidate genes in the region identified a T > A transversion in the third nucleotide of the codon encoding amino acid 106 of TYMS. This causes a predicted asparagine to lysine change. The structure and function of thymidylate synthase has been characterized extensively in various eukaryotes. The protein is a symmetric dimer, with each subunit ranging from 30 to 35 kDa depending on the organism. However, in protozoa and plants, TYMS and DHFR are produced on the same polypeptide (reviewed in [16]).

The main role of TYMS is to convert deoxyuridine monophosphate (dUMP) to deoxythymidine monophosphate (dTMP) using a 5,10-methylenetetrahydrofolate cofactor. dTMP is essential for the synthesis of DNA but not RNA, and therefore it is found at especially high levels in cells undergoing rapid cell division. Consequently, anti-TYMS drugs are used in cancer treatment, as are other chemicals that inhibit this pathway (such as methotrexate, a DHFR inhibitor) [17]. TYMS inhibitor drugs primarily target the dUMP substrate or the folate binding site on TYMS.

Since ASN106 is not specified as being a residue essential for TYMS function [18], we sought additional evidence that the mutation is responsible for the drastic phenotype of *L5Jcs11*. TYMS autoregulates its levels by binding its mRNA to decrease translation [19]. Consistent with a decrease in functional TYMS in cells bearing this allele, Western blots revealed that *L5Jcs11 *heterozygotes have ~2.6 fold elevated TYMS protein in the liver relative to WT mice (Figure 3b). Mutant heterozygotes also exhibited 2.3 fold lower DHFR protein levels (Figure 3b). ASN106 is located close to the folate binding motif of TYMS, suggesting that the enzyme is defective in binding or effectively utilizing the major substrate N_5_, N_10_-Methylene H_4 _folate.

To further explore the effects of this mutation on mice, we performed inner cell mass (ICM) outgrowth assays. As shown in Figure 3c, although trophoblast cells from cultured blastocysts could grow, consistent with the ability to implant, mutant ICMs were unable to outgrow from hatched embryos. This indicates that the rapid cell division and DNA synthesis occurring in embryonic cells cannot be supported without normal TYMS function, consistent with failed postimplantation development of the embryo proper.

#### *L5Jcs31 (Haus3)*

**Figure 5:**
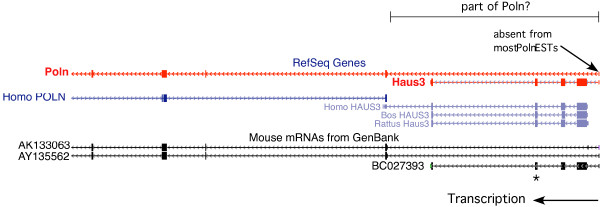
**Structure of *Haus3 *locus**. Shown are the following tracks from the UCSC browser (July 2007 NCBI37/mm9 assembly): Mouse RefSeq (red), Other Refseq (blue), and Mouse mRNAs (black). Refseq annotates *Haus3 *as being within the first intron of *Poln*, presumably based on the 2 "full length" mRNAs AK133063 and AY135562, which include a small 5' noncoding exon (far right). Interestingly, this exon was also found in a *Haus3 *full length mRNA as well as several spliced ESTs (not shown), but in only one spliced *Poln *EST (not shown). We posit that this 5' exon is not a normal part of *Poln *transcripts, but that occasional transcription initiated in that exon generally produced *Haus3 *mRNAs, but that occasionally there is readthrough to produce a *Poln *mRNA. Note that human, rat and cow *Poln *Refseq annotations do not include that exon, depicting the two genes as non-overlapping (with the exception of a potentially artifactually long 3' end of *HAUS3 *in the human). The *L5Jcs31 *mutation in *Haus3 *is indicated by an asterisk.

This mutation was found by high-throughput sequencing of hybrid-selected exon DNA. It is a T > C transition resulting in a LEU > PRO change in the fourth of 5 *Haus3 *exons. Currently, *Haus3 *is annotated as residing in the first intron of the *Poln *gene, in the same transcriptional orientation. However, this may be artifactual or not biologically relevant, since the putative exon 1 of *Poln *is: 1) non-coding; 2) shared with *Haus3*; 3) present in only 1 of >120 spliced ESTs aligned in the UCSC browser; 4) absent from human *POLN*; and 5) absent from other mammalian *Haus3 *genes (Figure 5). However, the recovery of "full-length" cDNA clones indicates that *Poln *mRNAs can be produced that contain this mRNA, as do many *Haus3 *transcripts (ESTs not shown). It remains to be seen if these two genes share a promoter/enhancer, or even a common transcriptional start site.

HAUS3 is a component of the multiprotein HAUS complex, homologous to the *Drosophila *Augmin complex. In mammalian cells, HAUS regulates centrosome and spindle integrity. Its disruption causes destabilization of kinetocore microtubules and centrosome disruption, and thus is critical for genome stability [20]. Interestingly, *HAUS3 *was found to be mutated early in the development of a subset of lobular breast cancers [21]. Though we haven't noted any obvious defects in heterozygous animals, this model might be useful for investigating the effects on breast cancer frequency or progression in tumor susceptible backgrounds. Homozygosity for *L5Jcs31 *causes pre- or peri-implantation lethality, indicating the essential nature of the augmin complex in mammalian cell growth or early development.

#### *L5Jcs32 (Fbxl5)*

This mutation was identified as a midgestation recessive lethal showing neural tube defects [9]. The allele was mapped by virtue of non-complementation with the *Hdh*^*df9J *^deletion. Sequencing of candidate genes in the region led to the identification of a T to A transition in exon 3 of *Fbxl5*, resulting in the replacement of methionine by lysine at amino acid position 127.

FBXL5 (F box and leucine-rich repeat protein 5) is part of an SCF (SKP1-cullin-F-box) ubiquitin ligase complex that plays a key role in regulating iron homeostasis [22,23]. It serves as a sensor of iron and oxygen levels by tethering the iron regulatory proteins IRP1/2 to an E3 ligase complex for ubiquitination and proteasome degradation, or alternatively, increasing transferrin receptor transcription and inhibiting ferritin production under conditions of low intracellular iron. The mutation resides in the conserved iron-binding hemerythrin domain of FBXL5, changing a highly conserved (invariant among vertebrates) MET immediately following a key histidine residue that is involved in coordinating Fe [22]. We postulate that this compromises or eliminates iron binding by the protein, thus disrupting its sensor function. Our findings that mutation of *Fbxl5 *causes embryonic lethality underscores the known critical nature of iron homeostasis during development (reviewed in [24]).

### The *L5Jcs13 *embryonic lethal allele maps to a gene desert

**Figure 6:**
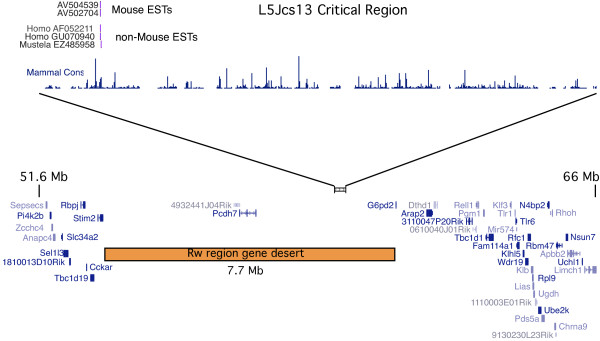
***L5Jcs13 *maps to a gene desert**. Shown are adaptations of UCSC browser displayed with selected tracks (July 2007 NCBI37/mm9 assembly). On top is the entire critical region to which *L5Jcs13 *maps (289 kb) with all annotated mouse and non-mouse ESTs. A graph of mammalian conservation (30-way Multiz alignment and conservation track) is indicated. The bottom half of the figure is a view of a larger region of Chr 5 in which the *L5Jcs13 *critical region is embedded. RefSeq genes are shown.

This mid-gestation embryonic lethal mutation was recombination-mapped to a 289 kb interval between SNP markers rs13478279 (60,593,878) and rs13478280 (60,882,847). No protein-coding genes or spliced ESTs are annotated in this region (Figure 6), nor are any known microRNAs. There appears to be an intronless, ORF-containing transcription unit (represented by two ESTs), with homology to transcripts in humans and weasel (Figure 6). However, no mutations were detected in the region spanned by the ORF in the *L5Jcs13 *allele (data not shown). We also sequenced an apparent processed pseudogene with homology to human POGK, and a conserved region corresponding to an unspliced EST EL606540, but again no mutations compared to the parental B6 sequence were found. Finally, approximately 59% of the critical region was scanned for mutations by shotgun sequencing of pooled PCR amplimers from *L5Jcs13/Rw *genomic DNA (see Methods). No *de-novo *mutations were found.

Remarkably, the *L5Jcs13 *critical region (289 kb) lies within a much larger gene desert that contains only 1 RefSeq gene (*Pcdh7*) in 7.7 Mb. The nearest gene is *G6pd2*, located 1.3 Mb distally, which marks the beginning of a gene rich region. The current data raises the possibility that the *L5Jcs13 *mutation affects a long range regulatory sequence, as appears to be the case with an ENU-induced allele of the quaking gene that is located 40-640 kb upstream [25], or the callipyge point mutation in sheep that affects transcription of genes up to several hundred kilobases away [26-28]. Alternatively, the mutation could disrupt the local chromatin structure, or interaction of chromatin with the nuclear matrix, in a manner that affects the expression of other genes. Finally, it is possible that there is an uncharacterized non-coding RNA in the critical region that is mutated. There is one reported case of a mouse ENU-induced mutation in a microRNA gene causing progressive hearing loss [29].

### *Ste5Jcs3 *maps to a region lacking mutated genes with mutations or implicated spermatogenesis roles

This spermiogenesis mutation was localized to a 1.6 Mb region (Figure 1) containing 4 annotated genes: *Tbc1d19, Rbpj*, *Cckar *and *Stim2*. We sequenced all of these (cDNA or exons from genomic DNA) and found no mutations. Additionally, the latter 3 have been mutated by gene targeting to produce phenotypes not consistent with specific male infertility. CCKAR-deficient mice are viable with no fertility defects [30,31]. Mutation of *Rbpj *causes embryonic lethality. *Stim2 *null mice are slightly runted and die by 4-5 weeks of age [32]. That study reported T cell defects but did not address germ cell development. *Tbc1d19 *appears to be widely expressed based on EST library representation (Unigene), and, like *Tbc1d14 *(mutated in *L5Jcs5*), encodes a likely Rab-Gap containing the TBC domain. Further sequencing analyses will be required to identify the mutation and determine if it affects a novel element, the expression of any of these genes, or the expression of genes located outside the genetically defined interval.

## Conclusions

This report summarizes work on a region-specific saturation mutagenesis project that was initiated before the mouse genome was sequenced. We reasoned that such regional screens (using deletions or balancer inversions such as *Rw*) provided powerful advantages for phenotype characterization and gene identification, including simplified stock maintenance using a single visibly marked balancer chromosome, and the knowledge of a mutation's location. Coupled with nested deletions generated in the same region [33], it was possible to rapidly sublocalize mutations to regions upon which to concentrate recombination mapping and guide efficient complementation analyses. The sum of this work was the molecular identification of 16 mutant alleles representing 13 genes. For 10 of these genes, the mutants are/were the first reported in mice (Table 1), and 6 are presented here for the first time. The remaining mutations have been mapped to relatively small intervals.

Perhaps the most interesting mutation thus far is *L5Jcs13*, which is a recessive embryonic lethal allele mapping to a gene desert. Clearly, this region contains a sequence element of crucial importance to mouse development, and close scrutiny of this region for evidence of cryptic genes (using *ab initio *gene prediction programs), highly conserved elements or transcription units have not revealed any candidate elements that contain mutations (data not shown). In an era where all protein-coding genes will soon be knocked out in ES cells by the KOMP project, the identification of apparently novel, non-genic, essential genomic elements such as *L5Jcs13 *underscores the continuing value of unbiased forward genetic screens.

The drawback of forward genetic screens in mice has been the cost and time involved in identifying causative mutations. For genome-wide screens, genetic crosses must be conducted in order to localize mutations, followed by traditional positional cloning efforts guided by candidate gene prioritization. Region-specific screens ameliorate the mapping part, and in one study the approach was taken to directly resequence all exons (and their flanking sequences) in the target region to identify causative mutations [4]. Potentially causative mutations were identified in 31 of the 41 mutant lines examined, and most of the potentially causative mutations were in non-coding regions within or near the transcription unit. The failure to identify the other 10 mutations amongst annotated genes, and the cases of *L5Jcs13 *and *Ste5Jcs5*, indicate that there is much we don't know about the functional content of the genome. Furthermore, we must caution that for the potentially causative mutations presented here and in other studies such as the aforementioned, functional validation must be obtained. This caveat also underscores the value of genetic mapping, which contributes to validation of a mutation identified by sequencing.

The advent of high-throughput DNA sequencing technologies can be transformative for forward genetics in mice. Here, we identified three mutations by Solexa/Illumina resequencing of exon-enriched DNA from critical regions. However, the rapidly declining cost of whole genome sequencing will soon negate the benefit of such sequence enrichment, or even genetic mapping in advance of sequencing (bearing in mind the issue of validation mentioned above). Additionally, whole genome sequencing has the important advantage of enabling the identification of non-coding regulatory elements.

## Methods

### Sequence capture and next generation sequencing

A custom Agilent oligonucleotide array was designed to sequence exons, UTRs and promoters of all RefSeq genes in non-recombinant intervals of several mutations (from this and unrelated projects). The array included all RefSeq genes between *D5Mit388 *and *D5Mit268 *(mm8; NCBI Build 36). Solution-based hybrid capture of *L5Jcs14*/+^B6^, *L5Jcs31*/+^B6 ^and C57BL/6J genomic DNAs to transcribed RNA oligonucleotide baits was performed as described [34]. Libraries made from captured DNA were sequenced using 51 or 76 bp paired-end reads with an Illumina Genome Analyzer. Candidate single nucleotide changes were identified by 1) aligning high quality sequence reads to the reference C57BL/6J mm8 genome sequence, 2) identification of single nucleotide polymorphisms (SNPs) between each sample against the reference, and 3) filtering for heterozygous SNPs unique to the mutant sample. In addition to Sanger sequencing, genotyping of *L5Jcs14 *SNPs with the Sequenom iPlex technology was performed to confirm the *L5Jcs14 *mutation.

For *L5Jcs13*, 70 primer pairs were developed to amplify the 289 kb critical region. The products averaged a length of 4.5 kb and overlapped approximately 400 bp on each end. Amplification was initially attempted on all products using Roche Expand Long Range Polymerase or iProof Polymerase (Bio-Rad). Out of the 70 primer pairs, 51 showed some level of successful amplification. Amplimers were treated with FastAP Alkaline Phosphatase plus Exonuclease I, pooled, then sequenced on an Illumina GA genome analyzer using chemistry that generated 43 nucleotide, single-end reads. The sequence was aligned to C57BL/6J ("B6") genomic sequence using Novoalign v2.07 and Stampy 1.0.9. Further comparison was done with the C3H/HeJ reference sequence from the Sanger Institute. Data was analyzed for single nucleotide polymorphisms (SNPs), insertions, and deletions using GATK v 1.4418 (The Genome Analysis Toolkit; http://www.broadinstitute.org/gsa/wiki/index.php/The_Genome_Analysis_Toolkit) and VarScan v 2.2.3 http://sourceforge.net/projects/varscan/. Candidate SNPs were compared to the NCBI SNP database dbSNP, and ultimately re-sequenced by standard methods to resolve whether a nucleotide difference was a SNP, a mutation, or a database or sequencing error.

### Generation of *Tapt1 *mice

ES cells containing a deletion of the entire *Tapt1 *locus were obtained from the KOMP Repository (clone name Tapt1_AG12, produced by Regeneron, Inc.). The targeted cells were of strain C57BL/6N, and the official allele name is *Tapt1*^*tm1(KOMP)Vic*^. Cells were microinjected into albino CD1 blastocysts to produce chimeras.

### Western blot analysis

Snap-frozen liver samples were lysed in 10 mM Tris, pH 7.4, 150 mM NaCl, 5 mM EDTA, 5 mM DTT, 1% Triton X-100, and Mammalian Protease Inhibitor Cocktail (Sigma). Tissue lysates were loaded onto 12% SDS-PAGE gels with 25 μg total protein/lane; protein concentrations were determined using the Lowry-Bensadoun method [35]. Proteins were then transferred to an Immobilon-P PVDF Membrane (Millipore). The membrane was blocked overnight at 4°C in phosphate-buffered saline with 10% nonfat dry milk and 1% NP40. The membrane was incubated for two hours at room temperature in one of two primary antibodies: 1:1000 -anti-TYMS (Zymed) or 1:2000 anti-DHFR (Sigma). After four washes (phosphate buffered saline with 0.1% Tween-20) of 10 minutes each, the membranes were incubated for 1 hour in 1:10,000 HRP-conjugated goat anti-mouse antibody for TYMS or 1:20,000 HRP-conjugated goat anti-rabbit for DHFR (Pierce). After four washes of 10 minutes each, membranes were developed in SuperSignal West Pico Chemiluminescent Substrate (Pierce) and films exposed. As a loading control, membranes were also probed with 1:100,000 HRP-conjugated anti-beta actin (Abcam). Quantification of protein bands was done by digitizing the films, then analyzing the images with ImageJ software (NIH).

### Blastocyst Outgrowths

Blastocysts (E3.5) were plated onto gelatinized tissue culture dishes, cultured for 5 days in ES cell medium, and harvested for genotyping as described previously [10].

### Mutation Mapping and screening

For recombination mapping, *Rw/L5Jcs# *animals were crossed to *C5Cast/C5Cast *(a strain in which the proximal portion of Chr 5 from *Mus castaneous *was rendered partially congenic in strain C3HeB/FeJ), and non-*Rw *F_1 _progeny (*L5Jcs#/C5Cast*) were intercrossed to produce F_2_'s. Genomic DNA isolated from tails from F_2 _animals were genotyped with polymorphic markers within the *Rw *region to identify recombinants. The locations of the mutations were determined indirectly, in that we excluded intervals in which a mutation resides by virtue of being able to obtain homozygosity for the parental B6 allele in live offspring.

Markers not in the MGD database are:

*D5Jcs640 *GCCAGGTTAATACAAGCTCCA and TCTCCTTCTTCCTTCTCTTCTCTTC

*D5Jcs85 *GGGCTTTTAGACGAGCAGAG and TGGGTTCAGAACGAAGGTCT

*D5Jcs24 *AAACATGTCAGGGCCAGAAG and TGTGCTTCCATTCATTTATGC

Mutation screening by dHPLC was performed on a Transgenomic Wave machine, designed to detect heteroduplexes. The screening was generally performed on heterozygous DNAs. Otherwise, amplified DNAs were sequenced by standard Sanger protocols in an automated sequencer.

All experiments using mice were conducted with approval of Cornell's Institutional Animal Care and Use Committee, protocol # 2004-0038.

## Authors' contributions

The following authors contributed to the mapping and/or positional cloning of one or more mutations: AKB, LMA, JYS, HKP, EKS, SLK, YC, RJM, WM, YL, KJS, TPH, MS, JM, DLR and JCS. LS conducted analysis of sequence data for *L5Jcs13*; JFG conducted Western blot analyses of TYMS and PJS interpreted the data on the *Tyms *mutation and wrote that section; JLM, KLT, EM, TF and FdP participated in the identification of mutations by sequence capture; JCS wrote the majority of the paper. All authors read and approved the final manuscript.

## Authors' information

AKB, LMA, JYS, HKP, EKS, DLR and SLK were undergraduates at Cornell when they conducted the research.
